# Serum peptidome patterns of breast cancer based on magnetic bead separation and mass spectrometry analysis

**DOI:** 10.1186/1746-1596-7-45

**Published:** 2012-04-20

**Authors:** Nai-Jun Fan, Chun-Fang Gao, Guang Zhao, Xiu-Li Wang, Qing-Yin Liu

**Affiliations:** 1 Institute of Anal-colorectal Surgery, No. 150 Central Hospital of PLA, No. 2, Huaxiaxi Road, 471000, Luoyang, China; 2 The Clinical Laboratory, No. 150 Central Hospital of PLA, No. 2, Huaxiaxi Road, 471000, Luoyang, China

**Keywords:** Breast neoplasms, Diagnosis, Proteomics, Matrix-assisted laser desorption/ionization time-of-flight mass spectrometry

## Abstract

**Background:**

Breast cancer is one of the most common cancers in the world, and the identification of biomarkers for the early detection of breast cancer is a relevant target. The present study aims to determine serum peptidome patterns for screening of breast cancer.

**Methods:**

The present work focused on the serum proteomic analysis of 36 healthy volunteers and 37 breast cancer patients using a ClinProt Kit combined with mass spectrometry (MS). This approach allows the determination of peptidome patterns that are able to differentiate the studied populations. An independent group of sera (36 healthy volunteers and 37 breast cancer patients) was used to verify the diagnostic capabilities of the peptidome patterns blindly. An immunoassay method was used to determine the serum mucin 1 (CA15-3) of validation group samples.

**Results:**

**S**upport Vector Machine (SVM) Algorithm was used to construct the peptidome patterns for the identification of breast cancer from the healthy volunteers. Three of the identified peaks at m/z 698, 720 and 1866 were used to construct the peptidome patterns with 91.78% accuracy. Furthermore, the peptidome patterns could differentiate the validation group achieving a sensitivity of 91.89% (34/37) and a specitivity of 91.67% (33/36) (> CA 15–3, *P* < 0.05).

**Conclusions:**

These results suggest that the ClinProt Kit combined with MS shows great potentiality for the diagnosis of breast cancer.

**Virtual slides:**

The virtual slide(s) for this article can be found here: http://www.diagnosticpathology.diagnomx.eu/vs/1501556838687844

## Background

Breast cancer is a leading cause of mortality and morbidity for women [1]. Surgery, chemotherapy, and radiation treatments can be effective, depending on stage of cancer and other factors [2,3]. At present, the best available tool for the early detection of breast cancer is mammography[4]. Data acquisition, processing and visualization techniques of medical images facilitate diagnosis and improve their functionalities [5]. However, it is well established that mammography is better able to detect certain types of breast cancer (such as ductal carcinomas) than other types (such as poor prognosis estrogen receptor (ER)-negative tumors)[6-9]. Considering ER status, interval detected tumors are 1.8 to 2.6-fold more likely to be ER-compared to screen detected tumors [8,9]. Combining findings derived from both cytology and histology best allows for the proper management of patients suffering from breast cancer [10,11]. However, cytology and histology testes are invasive, which are not suitable for screening of breast cancer. Continued improvements in our ability to detect breast cancer early offer the promise of further reducing the burden of this disease, as breast cancer detected at an earlier stage is much more curable than is metastatic disease.

Proteomics, which concerns comprehensive protein profile changes caused by multiple gene alterations, is currently considered the most powerful tool for the global evaluation of protein expression [12]. Human serum contains thousands of proteolytically derived peptides called peptidomes, which may provide a robust correlation with the physiologic and pathologic processes in the entire body [13,14]. Preliminary studies have shown that great interest has been focused on the low-molecular-weight region, particularly on peptides smaller than 20 kDa, which may provide a novel means of diagnosing cancer and other diseases [14-16].

Advances in mass spectrometry (MS) now permit the display of hundreds of small- to medium-sized peptides using only microliters of serum [17,18]. Matrix-assisted laser desorption/ionization time-of-flight MS (MALDI-TOF MS) can detect peptides with low molecular weights with the necessary sensitivity and resolution, which makes it a useful technique for serum peptide profiling. Furthermore, for accurate MS analysis, the peptidome fractionation procedure and the preanalytical conditions of peptidome mapping must be carefully assessed [19]. Magnetic beads (MBs), based on nanomaterials, have been developed and considered as a promising material for convenient and efficient enrichment of peptides and proteins in biological samples [20,21]. The combination of MALDI-TOF MS and MBs enables the high throughput and sensitive investigation of peptides and proteins.

In the current study, a novel technology platform called ClinProt (BrukerDaltonics, Ettlingen, Germany) was used. It comprises a immobilized affinity copper ions MB (IMAC-MB)-based sample separation, MALDI-TOF MS for peptide profiling, and a bioinformatics package for inspection and comparison of data sets to create “disease-specific” peptidome pattern models, which could serve as a powerful tool for breast cancer diagnosis [22-24]. The diagnostic model, which consists of three differentially expressed peptides, was established and validated by the Support Vector Machine (SVM) Algorithm, by which different groups were effectively discriminated. Then, the diagnostic model was further verified using blinded samples from breast cancer and healthy volunteers.

## Patients and methods

### Reagents and instruments

The AutoFlex III MALDI-TOF mass spectrometer, MTP 384 target plate polished steel, α-cyano-hydroxycinnamic acid (CHCA), MB-IMAC kit, and peptide calibration standard were purchased from BrukerDaltonics (Leipzig, Germany). The trifluoroacetic acid (TFA) and acetonitrile (ACN), and mucin 1 (CA15-3) diagnostic kit (ELISA) were purchased from Alfa Aesar (Ward Hill, MA, USA), Sigma (St. Louis, MO, USA), and Roche Diagnostics GmbH (SandhoferStrasse, Germany), respectively.

### Patients and sample collection

With their consent, 72 healthy volunteers and 74 breast cancer patients (TNM I 26, II48) were enrolled into the study, from whom blood samples were collected. Serum samples were prepared by collecting blood in a vacuum tube and allowing it to clot for 30 min at room temperature. About 1 mL of serum was obtained after centrifugation at 2000 rpm for 10 min and stored in small aliquots at −80°C until analysis.

### Study design

The data set, including 72 health subject and 74 breast cancer patients, was randomly split into model construction group and external evaluation group. Model construction group (36 healthy volunteers and 37 breast cancer patients) was used for the identification of signals related to peptides expressed differentially among breast cancer patients compared with healthy volunteers. The group was also used for the pattern recognition. The external evaluation group (36 healthy volunteers and 37 breast cancer patients) was used for the blind independent pattern validation of the cluster. The accuracy of the peptidome model was compared with that of CA 15–3. The mean ages (years, means ± SD) of the healthy volunteers and breast cancer patients were 56.35 ± 2.58 and 58.57 ± 9.55, respectively. The difference of ages between the healthy volunteers in the model construction group and those in the external evaluation group were not significant. No significant differences were also observed for the ages of the breast cancer patients and healthy volunteers, as well as for the TNM stages of the breast cancer patients in the model construction group and external evaluation group.

### Sample purification

IMAC-MBs were used for the peptidome separation of samples following the manufacturer's standard protocol [25]. First, 50 μL of IMAC-MB binding solution and 5 μL of IMAC beads were combined in a 0.5 mL microfuge tube after thoroughly vortexing both reagents. The microfuge tubes were then placed in an MB separator (MBS) and agitated 10 times. The beads were collected from the tube walls 1 min later. Then resuspend the MB in 20 μL of IMAC-MB binding solution. Second, 5 μL of serum sample was added and mixed by pipetting up and down. The microfuge tubes were then placed in an MBS and agitated 10 times. The beads were collected from the tube walls 1 min later and the supernate was carefully removed using a pipette. Third, 100 μL of IMAC-MB wash buffer was added into tubes, which were again agitated 10 times in the MBS. The beads were then collected from the tube walls, and the supernate was carefully removed using a pipette. After three times washing, 10 μL of the IMAC-MB elution buffer was added to disperse the beads in tubes by pipetting up and down. The beads were collected from the tube walls after 5 min, and the clear supernate was transferred into fresh tubes. The supernate was then ready for spotting onto MALDI-TOF MS targets and measurement. Finally, prior to the MALDI-TOF MS analysis, the targets were prepared by spotting 1 μL of the proteome fraction on the polished steel target (BrukerDaltonics, Bremen, Germany). After air-drying, 1 μL of 3 mg/mL CHCA in 50% ACN and 50% Milli-Q with 2% TFA was applied onto each spot, and then, the target was air-dried again (cocrystallization). The peptide calibration standard (1 pmol/μL peptide mixture) was applied for machine calibration.

### MS analysis

For proteome analysis, a linear Autoflex III MALDI-TOF mass spectrometer was used with the following settings: ion source 1, 20.00 kV; ion source 2, 18.60 kV; lens, 6.60 kV; and pulsed ion extraction, 120 ns. Ionization was achieved via irradiation with a crystal laser operating at 200 Hz. For the matrix suppression, a high gating factor with signal suppression up to 600 Da was used. The mass spectra were recorded in linear positive mode. Mass calibration was performed using the calibration mixture of the peptides and proteins in the mass range of 1–18 kDa. Three MALDI preparations (MALDI spots) were measured for each MB fraction. For each MALDI spot, 1600 spectra were quantified (200 laser shots at eight different spot positions). The spectra were recorded automatically using the Autoflex Analysis software (BrukerDaltonics, Bremen, Germany) for the fuzzy-controlled adjustment of the critical instrument settings to generate raw data with optimized quality.

### Bioinformatics and statistical analysis

The ClinProt Tools software 2.2 (BrukerDaltonics) was used to analyze all serum sample data derived from either the patients or the normal health subjects. The data analysis began with raw-data pretreatment, including baseline subtraction of spectra, normalization of a set of spectra, internal peak alignment using prominent peaks, and a peak-picking procedure. The pretreated data were then used for visualization and statistical analysis in ClinProt Tools.

Statistically significant differences in peptide quantity were determined using Welch’s *t*-tests. The significance was set at *P* < 0.05. The class prediction model was set up using the SVM Algorithm. Then, the classified peptidome patterns were constructed. To determine the accuracy of the class prediction, a cross-validation was first implemented. Twenty percent of the samples from the model construction group were randomly selected as a test set and the remaining samples were taken as a training set in the class predictor algorithm. Second, by designing a double-blind test, the samples of external evaluation group were classified using the classified peptidome patterns constructed by the SVM Algorithm.

### Detection of CA15-3

The serum CA15-3 levels of the 37 breast cancer patients and 36 healthy volunteers in the evaluation group were measured using an electrochemiluminescence immunoassay following the manufacturer’s standard protocols (the methods were omitted). The samples were diagnosed as breast cancer (≥ 50 U/mL) or healthy (< 50 U/mL).

### Statistical methods and evaluation of assay precision

Each spectrum recorded using the MALDI-TOF MS was analyzed with Autoflex Analysis to detect the peak intensities of interest and with ClinProt™ software (BrukerDaltonics) to compile the peaks across the spectra recorded from all samples. This setup allowed differentiation between the cancer and the health subject samples. To evaluate the precision of the assay, the within- and between-run variations were determined using multiple analyses of bead fractionation and MS for two plasma samples. For the within- and between-run variations, three peaks with various intensities were examined. The within-run imprecision was determined by evaluating the coefficient variations (CVs) for each sample, using eight assays within a run, and then the between-run imprecision was determined by performing eight different assays over a period of seven days. SPSS 16.0 was used to analyze the clinical characteristics of the volunteers using a χ^2^ test or a *t*-test. The significance was set at *P* < 0.05. In addition, SPSS 16.0 was used to compare the accuracies of the peptidome models and the CA15-3 determination.

## Results

For the reproducibility of the protein profiling, the within- and between-run reproducibility of two samples was determined via IMAC-MB fractionation and MALDI-TOF MS analysis. In each profile, three peaks with different molecular masses were selected to evaluate assay precision. Despite varying peptide masses and spectral intensities, the peak CVs were all <3% and <9% in the within- and between-run assays, respectively. These values were consistent with the reproducibility data for the Protein Biology System reported by BrukerDaltonics.

**Figure 1:**
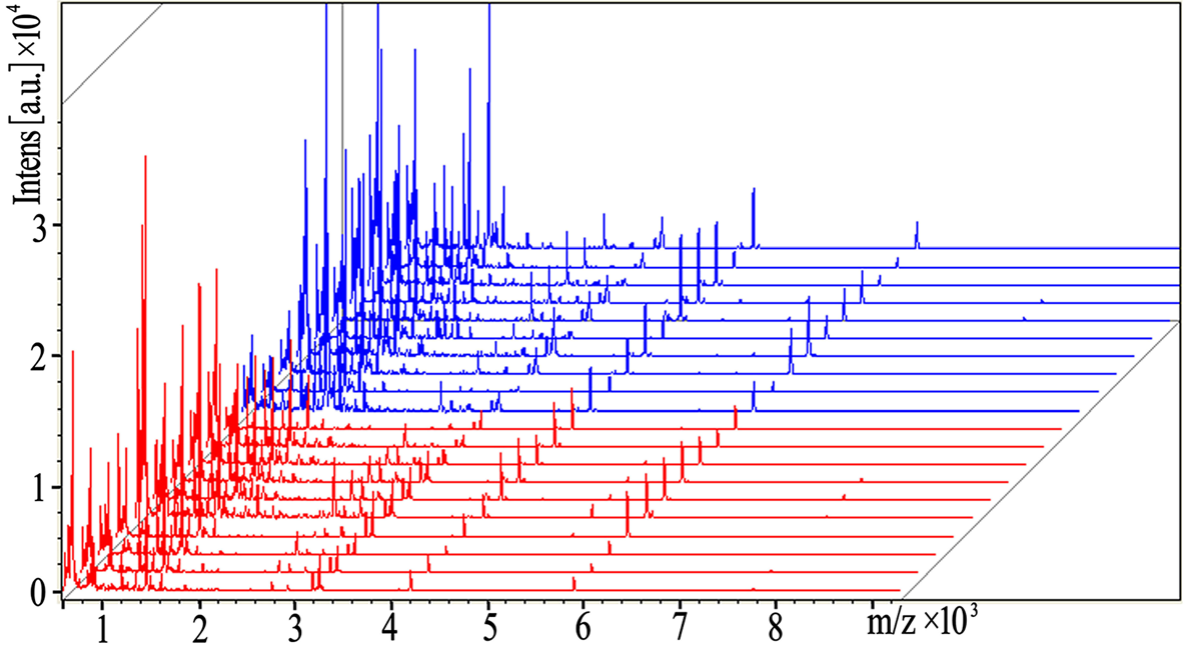
View of the aligned mass spectra of the serum protein profiles of the model construction group (red represents healthy volunteers, and blue denotes breast cancer patients) obtained using MALDI-TOF after purification with IMAC-MBs.

In the pilot study, the differences between the serum proteome profiles of breast cancer patients and healthy volunteers were evaluated. The mass spectra from 600 Da to 18 kDa were obtained using MALDI-TOF MS in linear mode. The representative mass spectra of the prefractionated sera of the model construction group are reported in Figure 1. On average, 70 signals common to the two groups have been detected in this mass range and 24 were identified by the ClinProt software with a statistically different area (*P* < 0.05 using the Wilcoxon analysis) in the model construction population, including 15 upregulated and 9 downregulated peptides, respectively (Table 1).

**Table 1:** Statistics of the 24 candidate biomarkers for identifying breast cancer patients from healthy individuals

**Mass**	**Breast cancer**		**Health**		**Regulation in breast cancer**	***P***^***★***^
	**Means**^**▲**^	**SD**	**Means**^**▲**^	**SD**		
622.48	48.9	24.43	34.29	18.28	↑	0.0454
622.97	48.26	24.67	30.41	14.76	↑	0.013
654.73	27.08	9.66	18.02	5.6	↑	0.0048
655.23	23.48	8.61	16.35	4.46	↑	0.00827
666.78	13.96	6.64	20.73	6.29	↓	0.00821
667.12	13.07	6.56	18.81	5.77	↓	0.0146
676.83	39.48	25.42	15.69	15.75	↑	1.98E-05
698.4	116.2	35.99	76.07	27.99	↑	0.0048
698.81^§^	114.32	35.83	70.97	23.34	↑	0.00138
720.8^§^	13.59	6.5	23.41	8.61	↓	0.00199
721.4	13.31	6.39	19.92	7.96	↓	0.0165
858.11	13.28	6.03	8.11	3.76	↑	0.0182
887.23	10.15	3.38	8.33	5.93	↑	0.0222
893.99	12.58	7.56	21.87	8.11	↓	0.00218
909.06	27.78	15.1	40.15	14.33	↓	0.0127
909.75	26.61	15.1	37.89	14.06	↓	0.0222
1618.46	11.12	8.56	21.88	16.05	↓	0.017
1866.64^§^	6.66	5.23	12.97	7.21	↓	0.00199
2770.34	6.64	5.11	2.26	1.07	↑	0.00123
2771.86	5.73	4.78	1.48	0.68	↑	0.000427
2933.97	3.69	2.06	1.86	0.85	↑	0.0103
5963.91	0.71	0.76	0.2	0.24	↑	0.0222
7772.04	0.73	0.94	0.25	0.46	↑	0.0176
7777.16	0.8	1.01	0.3	0.51	↑	0.0406

^§^The peptide selected for model construction.

^▲^Peak area.

^★^P value calculated with the Wilcoxon test; values lower than 0.05 suggest statistical relevance.

**Figure 2:**
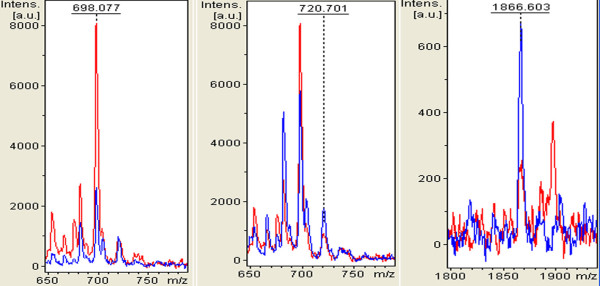
**Zoom of the mass range for the three signals (MALDI-TOF linear mode) used in the cluster to differentiate BREAST CANCER from healthy (H) individuals.** Red represents breast cancer patient, and blue represents healthy volunteers.

Classification models were developed to classify between the breast cancer and healthy volunteers samples of model construction group. The use of individual peaks as diagnostic biomarkers for breast cancer was addressed using SVM algorithm analysis. First, the breast cancer patients and healthy volunteers were compared. Second, all detected peaks were analyzed using ClinProt 2.2 to generate the cross-validated classification models. The optimized model resulted in the following correct sample classification. Three peptide ion signatures (m/z 698, 720 and 1866) were provided as a class prediction for a cross-validation set to discriminate the breast cancer patients from healthy volunteers, which achieved 91.78% recognition and 91.78% cross-validation accuracy. The regions of the mass spectra obtained at 800 resolution are reported in Figure 2.

**Figure 3:**
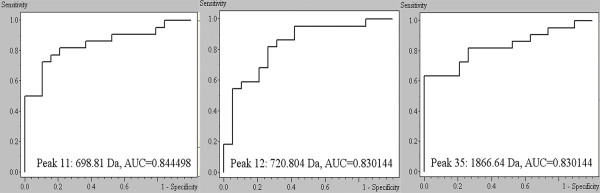
**The receiver operating characteristic (ROC) curves of the three signals selected for the diagnostic model**. AUC: Area under the receiver operating characteristic curve.

The preliminary statistical analysis was performed for each single marker and signal cluster using the receiver operating characteristic curve analysis. The area under curve (AUCs) of receiver operator characteristic (ROC)of peak A at m/z698, peak B at m/z 720 and peak C at m/z 1866 were 0.85, 0.83 and 0.83, respectively (Figure 3). Moreover, the areas of these peaks in the spectra of breast cancer patients were statistically different from those of the healthy volunteers (Figure 2). A combination of these three peaks yielded 88.89% (32/36) specificity and 94.59% (35/37) sensitivity for the breast cancer samples (Table 2).

**Table 2:** The predicted results of peptidome pattern distinguishing breast cancer patients from health volunteers

**Group**	**Cases**		**Sensitivity (%)**	**Specificity (%)**	**PV**_**+**_	**PV**_**−**_	**YI**
	**Breast cancer**	**Health**					
Model construction	37	36	94.59	88.89	89.74	94.12	0.83
External validation	37	36	91.89	91.67	91.89	91.67	0.83

Abbreviations: *PV*_*+*_, Positive predict value; *PV*_*−*_, Negective predict value; *YI*, Youden index.

To verify the accuracy of the established SVM classification model with the adopted peptides, the samples of external evaluation group was introduced (not used in the model construction), which consisted of 37 breast cancer patients and 36 healthy subjects. As a result, the model correctly classified 91.89% (34/37) of the breast cancer (sensitivity) and 91.67% (33/36) of the healthy (specificity) samples, which surpassed the results of CA15-3 (41.67% (15/36) specificity and 43.24% (16/37) sensitivity) (Table 2).

## Discussion

The usefulness of multiple markers for diagnosis, prognosis, and prediction of the risk of developing diseases or their complications is now widely recognized [13,26]. Various proteomic approaches have been applied to biomarker discovery using biological fluids. Interestingly, low-molecular-weight peptides, such as S100A8 and fibrinogen, have been recognized to play important roles in physiologic and pathologic processes and could be used as relevant biomarker candidates [27,28]. Recently, the mass spectrum that directly detects and differentiates short peptides has offered a promising approach for peptidomic biomarker discovery [14,15,29-31].

MS instrumentation and analysis tools have continued to rapidly evolve and improve our ability to detect lessabundant serum proteins. Until now, the most commonly used instrument was the SELDI-TOF MS[32-35]. However, SELDI-TOF MS does not allow a direct identification of the discriminatory proteins and the debate about the reproducibility has been particularly strong[36]. Alternative approaches for measuring polypeptides, such as the surface-enhanced laser desorption and ionization, recently reported by several groups, have several disadvantages, such as low resolution and the loss of most proteins and peptides [37-39]. MALDI is a soft ionization technique used in MS that allows the analysis of biomolecules such as proteins, peptide sugars, and large organic molecules. As a powerful tool for surveying the complex patterns of biologically informative molecules, MALDI-TOF MS protein/peptide profiling has been applied in proteomics biomarker research and has become a promising tool in cancer biomarker research [29,40,41].

In the present study, by integrating short peptide purification with IMAC-MBs, peak intensity detection with MALDI-TOF MS, and profile analysis with ClinProt Tools software 2.2, a series of differentially expressed short peptides in the sera of breast cancer patients has been successfully detected. A comparative case control analysis between breast cancer and healthy volunteers was performed. Peptidomic maps associated with the disease were drawn. The results show that compared with the healthy volunteers, the breast cancer patients share 24 significantly differentiated peptides, including 15 upregulated and 9 downregulated peptides. Genomic and proteomic technologies will further help us understand the intracellular signaling and gene transcription systems, as well as the protein pathways that connect the extracellular microenvironment to the serum or plasma macroenvironment of cancer [42]. These 24 interesting significantly differentiated peptides may provide further evidence for understanding the occurrence and progress of breast cancer.

Using SVM algorithm analysis, classification models were developed to classify samples between healthy volunteers and breast cancer. A cluster of three peptides at m/z 698, 720 and 1866 achieved a recognition capacity and a cross-validation of 91.78% to discriminate breast cancer from healthy volunteers. The blinded verification of the SVM classification model proved the correct classification of 91.89% (34/37) of the breast cancer (sensitivity) and 91.67% (33/36) of the healthy volunteers(specificity). To our knowledge, this is the first study to screen for breast cancer-related short peptides in sera by combining IMAC-MBs and MALDI-TOF MS. The classification model that we have built up has potential applications in providing alternatives for breast cancer diagnosis and may provide a better understanding of breast cancer pathogenesis or help in tailoring the use of chemotherapy for each patient, finally resulting in improved patient outcomes.

In conclusion, peptidome patterns from IMAC-MB-purified serum samples were directly profiled with MALDI-TOF MS and a peptidome model that differentiated breast cancer from the healthy volunteers was constructed with high sensitivity and specificity. Despite the high sensitivity and specificity, the number of specimens analyzed in this study was relatively small, which may limit the validity of the results. The next step in our study will be to analyze larger patient cohorts and to run blinded samples to confirm the usefulness of the currently identified peptides for breast cancer diagnosis. After this confirmation, the biomarkers of the interest will then be isolated and identified and their biological role in breast cancer pathogenesis will be studied.

## Competing interests

The authors declare that they have no competing interests.

## Authors’ contributions

N-JF carried out magnetic bead separation and mass spectrometry analysis, participated in the design of the study, and drafted the manuscript. C-FG conceived of the study, and participated in its design and coordination and helped to draft the manuscript. GZ participated in mass spectrometry analysis and performed the statistical analysis. X-LW carried out the clinical sample and data collection. Q-YL carried out the immunoassays. All authors read and approved the final manuscript.
